# Surgery

**Published:** 1861-11

**Authors:** John H. Packard

**Affiliations:** Philadelphia


OR, BI-MONTHLY NOVELTIES IN MEDICAL, PATHOLOGICAL, SURGICAL, OBSTETRICAL PHYSIOLOGICAL, AND CHEMICAL SCIENCE.

### SURGERY.

BY JOHN H. PACKARD, M.D., OF PHILADELPHIA.

I.—*Pulsating Tumors in the Orbit.* By Joseph Bell, M.D., L.R.C.S., etc. (Edinburgh Medical Journal, June, 1861.)

A detailed history of a case of this kind, successfully treated by Mr. Syme, at the Edinburgh Royal Infirmary, is given by Dr. Bell, who thus analyzes it: "A young woman, previously healthy, is suddenly seized with pain in the head, followed by an equally sudden snap, or crack, inside, resulting from no injury, but causing great deformity. A noise in her head, audible to herself and others, loud and persistent, renders her life miserable by its weary sameness, and the confusion of ideas which it creates. The tumor is rapidly increasing; vision, first rendered double, begins to fail entirely; the walls of the orbit are absorbed, and the patient's health rapidly undermined. Palliatives are of no use. Medical aid has failed. The carotid is tied; a perfect cure results; truly a triumph of surgery."

The pathology of this affection is less settled than the indications for its treatment. Dr. Bell seeks to throw light on the former point by consulting the symptoms and results of treatment in the recorded cases, and the post-mortem appearances in such as proved fatal.

(1) *Previous Cases.*—These are about twenty in number. Five of them correspond in every particular to that given by Dr. Bell; of these, two are reported by Mr. Nunneley, of Leeds, in vol. xlii. of the London *Medico-Chirurgical Transactions;* one by Mr. Travers, of London, in vol. ii. of the same; one by Mr. Dalrymple, of Norwich, in vol. vi. of the same; and one by M. Jobert, of Paris, in the *Mémoires de l'Acad. Royale de Médecine,* vol. ix.

"In very minute particulars these six cases correspond. Their principal characteristics are the following: The ages of the six varied from 22 to 65 years, but in all the health was weakened or impaired previous to the attack. Three of the five females were pregnant when the disease commenced; of the others, one was in delicate health, and the last was well advanced in years. M. Jobert's case was a bronchitic gentleman, 60 years old. In every one of the six cases, the suddenness of the pain was most remarkable; and in four of them, a loud noise, like the discharge of a gun, or the crack of a whip, was most distinctly heard and described by the patients, 'as if something' (to use the words of one) 'had given way inside of the head with a sudden snap;' and in all the cases this occurred spontaneously, without any blow, fall, or great exertion. This is a very remarkable symptom, and distinguishes these six cases from another set, which resemble them in many other respects. In all these six cases, the eyeball was protruded, and there was a pulsation communicated to it. In all six there was a well-marked bruit, described differently, as 'bellows-murmur,' 'rippling,' 'buzzing,' and 'muffled engine-stroke.' In all the pain was intense; in all the vision was impaired; in two partially, in four completely gone in the affected eye. In every case the eyelids were puffed and œdematous, and the conjunctival vessels injected. This is an important symptom, as tending to prove the existence of some obstacle to the venous return. In all six cases the common carotid was ligatured on the affected side; and the operation was followed in five cases by a complete cure of the disease, and in the other, by remarkable head symptoms, secondary hemorrhage, and death, though the immediate effect of the ligature in it also was to relieve the symptoms. The morbid appearances in the fatal case will be noticed hereafter.

"The cases of Travers and Dalrymple were called by their authors 'Cases of Aneurism by Anastomosis of the Orbit,' and as such they have been quoted by all succeeding writers on the same subject, until Mr. Bush, in the 22d, and Mr. Nunneley, in the 42d volumes of the *Medico-Chirurgical Transactions of London*, disputed the accuracy of this pathology, and substituted the name of 'Aneurism in the Orbit,' and 'Aneurism of the Orbit,' respectively. M. Jobert's case is called an aneurismal tumor, and is said to have had the 'susurrus' heard in varicose aneurisms; but of the existence of a varicose aneurism there is no evidence, nor from the result is there the slightest probability.

"Another series of cases of pulsating tumor in the orbit, while resembling each other very closely, differ in several important particulars from the first series. These are reported by Messrs. Bush, Scott, Curling, Velpeau, and Nunneley. These all occurred in males of middle age; and in the history of each, its origin was traced to some distinct and severe injury—either a blow or a fall—thus differing from the first six, which were all of spontaneous origin."

In all the cases of this second series except one, the protrusion of the eyeball and the pulsation increased very gradually, and none of the patients could give the exact date when he first noticed either pain or swelling. In all, ligature of the carotid was successful; in one, however, only for a time.

(2) *Post-mortem Appearances.*—Only two fatal cases can be adduced; one under the care of Mr. Nunneley, the other under that of Mr. Bowman.

"In Mr. Nunneley's case, the symptoms were particularly well marked. Sudden crack, as of a gun, when the patient was stooping; great pain and buzzing in the head; protruded eyeball and swollen lids; sight gone; distinct bruit, and decided pulsation. The artery was tied, with immediate cessation of the symptoms. The patient died sixteen days after the operation, and the following was found: 'The ophthalmic artery was considerably dilated; its coats thickened with atheromatous patches; two of its branches, particularly the inner, or the continuation of the trunk toward the inner angle of the orbit, were distended and filled with coagulum; the outer, or lachrymal branch, was also large, and filled with coagulated blood, but not to the same extent as the inner. Before the operation, the bruit and pulse were most decided on the inner side. All the other branches, arteries and veins were so small as hardly to be observed.' No sign there of aneurism by anastomosis; and the dilated arteries with atheromatous patches, in a woman aged 65, were very much what might have been expected, even had there been no pulsation in the orbit. Outside the orbit, however, the following was found, the importance of which seems to have been underrated: 'The left carotid, on emerging from the bony canal at the origin of the ophthalmic artery, was decidedly enlarged, and filled with and surrounded by a nodule of coagulum about the size of a large horse-bean; the right carotid was normal.'

"In Mr. Bowman's fatal case, (not however reported by Mr. Bowman,) which seems to have been a well-marked one, and the symptoms of which were at once removed by the ligature, the report of the pathology is very shortly given. 'At the autopsy no aneurism was found to explain the symptoms present during life.'

"It is indeed very remarkable that in these two cases, in which the symptoms were well marked, the operation performed so far with success, and examinations obtained, really nothing to account for the symptoms was discovered in the orbit. Where, then, are we to look for the cause? In default of a better explanation, and with the greatest possible deference to the recorded opinions of such able observers as Travers, Dalrymple, and Nunneley, I am inclined to think that the aneurismal condition of the internal carotid, so well described by the latter surgeon, is a fact of the very greatest importance, and may, in part at least, account for the symptoms. I do not say in all cases of pulsating tumors of the orbit, but in some of them, especially in such a one as Mr. Nunneley's, where really nothing else was found. In it we have extensive pulsation and protrusion to account for; yet all we find within the orbit is a slightly dilated condition of a small arterial branch, while the other arteries and veins are hardly visible. And it must be remembered that, as the fatal termination occurred on the sixteenth day after the operation, in that period there was no time for any previously existing aneurismal tumor to disappear. Might not the condition of the internal carotid account for the symptoms? The coagulum, large as a horse-bean, must have occupied space which was formerly completely filled; the bony walls of the cranium cannot yield; the coagulum and dilated artery must find room—at the expense of what parts? The vessels which are close by must be compressed, especially the ophthalmic vein; for the artery is more protected in its foramen, than the vein in the sphenoidal fissure. The compressed vein prevents the return of the blood from the orbit, accounting for the venous congestion and injection specially noticed in every case. This, too, explains, as the theory of aneurism by anastomosis can never do, the suddenness of the pain, and the rapidity of the protrusion. In a case reported by Sir Gilbert Blane, dilatations of the internal carotids, much smaller than the one in Nunneley's case, produced the most violent symptoms—acute pain, noise, and blindness. In another, by Professor Dudley, the diagnosis was an endocranial aneurism, while the symptoms of pulsation and protrusion in the orbit closely resembled the present case; the carotid was tied with success. What is the cause of the disease? *Aneurism by anastomosis* must be rejected on these grounds: 1. It could never account for the suddenness of the symptoms, which is so remarkable a feature of nearly all the cases. 2. Aneurism by anastomosis is *not* curable by ligature of vessels. * * * * 3. In the cases examined after death, no aneurism by anastomosis was found.

"*Aneurism, true or false, in the Orbit.*—This may certainly be considered as the cause in some of the cases. * * * Aneurisms in the orbit, though rare, are to be found in the records of pathology. Mr. Guthrie records one case in which there was protrusion of both eyes, with a hissing noise in the head, and in which aneurisms of both ophthalmic arteries, as large as nuts, were found on dissection. Mackenzie also quotes two cases of aneurism of the arteria centralis retinæ—one with pulsation in the orbit, besides loss of sight.

"*Obstruction to venous return in the sphenoidal fissure, by endocranial aneurism, or other tumor*, may be allowed as a cause, at least, in the cases where no true aneurism could be found on dissection, and especially in those cases occurring after blows on the head, and in connection with other head-symptoms denoting internal mischief."

II.—*On the Circumstances of the Regeneration of Bone.* By M. SÉDILLOT. (Gazette Hebdomadaire, Aug. 23, 1861.)

In a letter to the President of the *Académie des Sciences*, in Paris, M. Sédillot remarks that he has, in several of his previous communications, endeavored to show that failures in the regeneration of bone occur at points where the periosteum has been attacked with suppurative inflammation; and he would thus explain the formation of cloacæ. Such facts prove the risk involved in dissecting up the periosteum from bony surfaces, since the almost inevitable suppuration will prevent the renewal of the bone, the embryonic cells being converted into pus-globules instead of into osseous tissue.

After detailing a case of necrosis of the femur, the author draws the following conclusions:—

1. Operations are preferable which do not disturb the relations of the periosteum with the subjacent bone. 2. Operations are objectionable in which the periosteum is dissected up or isolated. 3. Reformation of bone cannot be looked for from the periosteum detached from splinters at the seat of a fracture. 4. Nor from a "cuff" of periosteum left at the extremity of a bone in amputating a limb. 5. Nor in cases of pseud-arthrosis treated by resection, the periosteal sheath being preserved. 6. Nor in ordinary resections of the bones of the limbs, flaps of periosteum being isolated and retained in the wound.

III.—*Osteo-plastic Resection of the Upper Jaw.* (Foreign Correspondence of the London Medical Times and Gazette, Sept. 7, 1861.)

This operation was done by Professor Langenbeck upon a boy fifteen years of age, on account of a fibroid growth springing from the left pterygo-palatine fossa, and extending not only into the spheno-maxillary fossa, but also into the cavities of the nose and pharynx.

Chloroform having been administered, an incision was made from the left ala nasi, through the tissues of the cheek, to the middle point of the lower edge of the zygomatic process of the temporal bone. Another incision was then carried from the nasal process of the frontal bone, along the lower margin of the orbit, so as to meet the previous one at its posterior extremity. The first incision was now deepened so as to divide the subcutaneous tissues, the masseter muscle, and the buccal fascia. A lobular, white, shining tumor now became visible, extending between the external surface of the upper jaw and the coronoid process of the lower. The two jaws being now separated, the finger could be carried in between the upper jaw and the tumor to the pterygo-palatine fossa. The pressure of the tumor had so enlarged this fossa, as well as the spheno-maxillary, that an elevator could now be readily passed into the pharynx. A fine, straight, narrow saw was now passed in, and the upper jaw divided horizontally, the septum narium being protected from the point of the saw by the left forefinger, introduced from the mouth. The second incision was now deepened to the bone, and the saw carried through the zygomatic process and the orbital process of the upper jaw to the lachrymal bone. Nothing now held the resected portion of the upper jaw except its connection, by its nasal process, with the frontal and lachrymal bones; and this allowed of its being raised by means of an elevator, like a box-lid. In this way the spheno-maxillary and pterygo-palatine fossa, and the naso-pharyngeal cavity, were rendered easily accessible; and the tumor, which had no connection with the base of the skull, could be readily and wholly removed, the part situated within the pharynx being drawn out with a pair of strong forceps.

A good deal of hemorrhage occurred during the operation, on account of the dilatation of the arteries; but it ceased spontaneously. One ligature was applied, to the spheno-palatine or nasal artery, and the thread brought through the left nostril. The wound was then carefully cleaned, and the jaw replaced. Some pressure was necessary in order to keep the bone in place, and the edges of the skin were brought together by means of iron-wire sutures. A cerate dressing was applied, with a pad of charpie over it, and a bandage to keep everything in place.

The case subsequently went on very well. In a fortnight the wound had healed, and the appearance of the face was nearly normal; although there was imperfect paralysis of the orbicular muscle of the left eye, and anæsthesia of the parts supplied by the infra-orbital nerve and the derivatives of the spheno-palatine ganglion.

IV.—*Luxations of the Upper Extremity of the Radius.* By MM. BERNADET and BOUVIER. (Gazette Hebdomadaire, Aug. 23, 1861.)

M. Bernadet lately exhibited to the *Société de Chirurgie* of Paris a specimen taken from the body of a man aged seventy-two, showing a luxation backward and outward of the upper end of the radius. The lesion was of long standing, having been sustained by a fall on the hand when the patient was but twelve years of age. No attempt at reduction had ever been made. All the movements were free except that of supination, which was imperfect.

As was remarked, in connection with this specimen, by MM. Bauchet, Morel-Lavallée, and Demarquay, luxations of the radius backward are of easy reduction when recent, but can be remedied only with great difficulty, if at all, when they are old. M. Désormeaux added, that even after reduction it was very hard to retain the bones in place; he had seen the lesion reproduced. The degree to which the functions of the elbow-joint are maintained, in spite of luxation of the radius, is well known.

M. Bouvier exhibited at the succeeding meeting a patient in whom this integrity of function was remarkably displayed. The man, thirty-nine years of age, had sustained a luxation backward of the radius in each arm, so early that he thought the lesion a congenital one. All the movements of the joint were in this case very free. Extension and flexion were normal, or at least were within ten degrees of being complete. Pronation was perfect. Supination was slightly limited, the forearm describing in this motion two-thirds of a semi-circumference instead of a whole one. The power of the arm was very little impaired, the man easily performing his functions as a hospital steward.

The interest of this case is great, in reference to its etiology. Were these luxations congenital? Such was M. Bouvier's belief, in opposition to that of M. Malgaigne, who denies that luxations are ever congenital, because they have never been observed in the fœtus. M. Bouvier acknowledged that they were very rare in the fœtus, but thought M. J. Guérin had seen one, although he has not given a very exact description of it. The existence of a double lesion is in favor of the idea that it is congenital.

V.—*On the Reduction of Dislocations.* By PROF. BUSCH. (Foreign Correspondence of the Medical Times and Gazette, June 22, 1861.)

At a recent meeting of the Society of the Lower Rhine, Professor Busch gave an account of some experiments made by him in regard to the reduction of dislocations without traction. Having to treat a case of luxation of the head of the humerus, he tried powerful traction unsuccessfully, the patient being under the influence of chloroform. "As in this case the impediment was probably of the capsular ligament, and the place where it was torn was known from the origin of the dislocation, after the patient had recovered from the effects of chloroform, reduction was tried by making the cleft of the capsular ligament open by a simple lever-movement of the arm. After this had been done, rotation of the arm was sufficient to bring the head of the os humeri back into the glenoid cavity. The facility with which reduction was thus accomplished, while the most powerful traction had been of no avail, was so striking that a series of experiments on dead bodies was made, in which dislocations of the humerus and of the femur were artificially produced. In each of these dislocations, in which considerable traction was necessary to remove the dislocated head of the bone only a little way from its wrong position, reduction was always very easily accomplished by rotation of the limb, provided the following rule was adhered to: The limb must be brought into that position, in relation to the trunk, in which it was at the time of the origin of the dislocation, and the upper edge of the cleft of the capsular ligament must be removed from the lower one (or the anterior from the posterior one) by the head itself, so that the cleft of the capsule becomes open. For instance, in dislocation of the femur backward, the surgeon ought to stand on the sound side, and take hold of the dislocated limb by the heel; he should then make extension in the knee-joint, and flexion in the hip-joint to a right angle, or even beyond that; after this he should adduct the limb, and then rotate it outward. If, in artificial dislocation, the parts are laid bare at the posterior side, it is seen that the head of the bone is, by the flexion, removed from that position in which it was after the dislocation had been produced, and brought closer to the edge of the acetabulum, while by the adduction it is raised over the posterior edge of the acetabulum, so that the surgeon can put his finger between this and the head of the os femoris, so that rotation outward is sufficient for establishing complete reduction. The success of this method was most striking in the different forms of dislocation of the femur; and it is probable that, in the living man, it will have to be chiefly employed in cases where the position of the limb, in relation to the trunk at the time of the origin of the dislocation, and the position of the cleft of the capsular ligament, are known. Perhaps it will also be of use in old dislocations, as by making the cleft of the capsular ligament open, pathological productions, by which it has been narrowed, may be broken. Professor C. O. Weber has been led by his experiments to the same conclusions."

VI.—*Substitute for Cerate as a Dressing for Wounds.* By M. DÉSORMEAUX. (Parisian Correspondence of the Lancet, June 29, 1861.)

This dressing was exhibited by M. Désormeaux at a recent meeting of the *Societé de Chirurgie:* he obtained the formula for it from the dispenser at the Hôpital St. Antoine. Its chief feature is the absence of fatty ingredients, which are replaced by glycerin, properly thickened by the addition of starch. About one part by weight of starch to eight of glycerin is the best proportion. Boiling water is poured on the starch till it attains a clear, pasty, fluid consistence; the glycerin is then added, and the whole rubbed up for ten minutes in a mortar. M. Désormeaux has found this preparation wholly unirritating.

VII.—*On Cysts within the Femoral Canal simulating Femoral Hernia.* By CHRISTOPHER HEATH, F.R.C.S., etc. (Med. Times and Gazette, Sept. 7, 1861.)

Mr. Heath alludes to the fact that these cases are very rare, the only ones he has found recorded being those mentioned by Mr. Quain in the *Medical Times and Gazette* for Jan. 6, 1855, ("On some Unusual Circumstances met with in Operations for the Relief of Strangulated Hernia,") by Mr. Chevalier in the *Medico-Chirurgical Transactions*, vol. iv., and by Breschet, as occurring in Dupuytren's practice.

Two such cases came under Mr. Heath's notice within the space of two years. One was in a woman aged 70, operated on for a small femoral hernia on the left side; death from peritonitis ensued in forty-eight hours, and an autopsy showed that the returned mass was "a cyst evidently derived from the peritoneum, though now shut off from it, and containing, apparently, a mass of *appendices epiploicæ* derived from the large intestine, the malposition of which" (it was close to the femoral ring) "was no doubt congenital. On laying open the intestine, a slight puckering of the mucous membrane was visible opposite the attachment of the cyst. There was not the slightest trace of peritonitis near the seat of the operation, and the case must be regarded as one of unrecognized enteritis, complicated by the presence of a tumor in the seat of hernia."

The other case was that of a woman aged 35, who had been treated for a supposed hernia several times. Symptoms of strangulation being present, and the taxis failing, an operation was resorted to. "Upon dividing the layer which appeared to be the sac, I found that the contained tumor was too transparent for gut, drew it carefully out, and found it to be translucent, and clearly a cyst. I carried the finger to the upper part, to see if it was connected with the bowels, and with the nail tore through the tissues which seemed to form a neck for it, and was thus enabled to isolate and remove it. It was a cyst some two inches long and three quarters of an inch in diameter, constricted in the middle, and containing clear, amber-colored fluid, having apparently no connection with the peritoneum or intestine, but distinctly occupying the crural canal. I put in a couple of stitches and applied a bandage.

"The patient recovered without a bad symptom."

VIII.—*On Lithotomy in Children.* By MR. FERGUSSON. (Medical Times and Gazette, May 18, 1861.)

In connection with the case of a boy three years of age, operated on at King's College Hospital, Mr. Fergusson remarked that he preferred to introduce as large a staff as possible, since it did not matter about its being readily movable, as in the case of the sound. The introduction of a small staff required more care and skill, in order to know where the point of the instrument pressed. The point of a small staff might pass out of the urethra, and this, too, although its general direction appeared to be correct. This, he said, was not a mere theoretical objection, as he had known such an accident to happen in the hands of a very distinguished operator. Mr. Fergusson added, that if in the case in which he had just operated, he had not touched the stone with the staff, he would not have proceeded. In reference to lithotomy in children, he said it was a rule with him to take more time and care than with adults, as he felt certain that there was much more risk of getting wrong in the former. In children, the tissues were less firm, and the resistance was therefore less. The incision also was required to be on a smaller scale. He always endeavored to make as small an opening as possible into the membranous portion of the urethra, and only just notch the prostate, or leave it altogether untouched. The next step, the introduction of the finger into the urethra, was, however, the one in which the greatest mischief might be produced by want of care—mischief often irreparable. The membranous portion of the urethra, and the neighboring tissues, were in children easily torn, and the urethra might give way above as well as below the staff, so as, separated all round, to be pushed before the finger toward the bladder. The finger would then move about in the pouch thus formed, and the completion of the operation would be almost impossible. He had, however, known a surgeon retrieve himself even after this error, which he thought he himself had once committed; in that case, it took him some time to get his finger into the bladder.

Mr. Fergusson decidedly prefers the lateral method of operating; he has employed it in forty cases of children, with success in all but one.

IX.—*On In-growing Toe-nail.* By M. WAHU, of Nice. (Boston Medical and Surgical Journal, Sept. 19, 1861, from the Gazette des Hôpitaux.)

M. Wahu had long suffered from this affection, and had tried many remedies, among them alum and Vienna paste, in the hope of avoiding the necessity of the surgical removal of the nail. He was at length struck with the idea of drying up or tanning the diseased surface, and decided upon the perchloride of iron as the most efficient means for so doing. "I obtained some," says he, "in a powdered form, and insinuated it as deeply as possible between the free edge of the nail and the ulcers. I felt almost immediately a moderate sensation of pain, accompanied by a feeling of constriction and a strong burning sensation. A quarter of an hour after I attempted to walk, and to my great satisfaction I found I could bear my weight on my foot throughout its entire length, without the least pain; a thing which I had not done before for many months. The following day I carefully examined the diseased parts, and found them mummified and as hard as wood. I applied a fresh quantity of the perchloride, which I allowed to remain a quarter of an hour, but I have reason to believe that this application was useless, as the mummification was complete by the first process. I continued to walk without the least thought of my *ongle incarné*, and about three weeks after was able, by means of a foot-bath, to remove the hardened layer of skin, under which I found a tissue of new formation, which perfectly resisted the pressure of the edge of the nail. Shortly afterward, the whole had returned to its normal condition, and since more than two years have passed without a return of the disorder."

